# Long-term effects on rate of torque development and fear of falling following high-speed resistance training in older adults

**DOI:** 10.1038/s41598-025-09095-8

**Published:** 2025-08-09

**Authors:** Alexandre Duarte Martins, João Paulo Brito, Orlando Fernandes, Bruno Gonçalves, Rafael Oliveira, Nuno Batalha

**Affiliations:** 1 Universidade de Évora, Comprehensive Health Research Centre (CHRC), Escola de Saúde e Desenvolvimento Humano, Departamento de Ciências Médicas, Largo dos Colegiais 2, 7004-516 Évora, Portugal; 2 Life Quality Research Center (CIEQV), Santarém Polytechnic University, Complexo Andaluz, Apartado 279, 2001-904 Santarém, Portugal; 3 Santarém Polytechnic University, School of Sport, Av. Dr. Mário Soares, 2040-413 Rio Maior, Portugal; 4 Research Center in Sport Sciences, Health and Human Development (CIDESD), Santarém Polytechnic University, Av. Dr. Mário Soares, 2040-413 Rio Maior, Portugal

**Keywords:** Aging, Physical activity, Exercise, Neuromuscular monitoring, Muscle contraction, Ageing, Neurophysiology, Geriatrics, Lifestyle modification

## Abstract

This study examined the influence of physical activity (PA) on the retention of rate of torque development (RTD) effects achieved following a 16-week high-speed resistance training (HSRT) over a 12-month follow-up period. Secondly, the fear of falling was also assessed. After the follow-up, 36 participants were categorized into two groups according to the PA: light activity group (LAG, *N* = 20, age 70.00 ± 3.66 years) and moderate-to-vigorous activity group (MVAG, *N* = 16, age 68.50 ± 2.09 years). At the four time points, RTD_PEAK_ and its time intervals for knee extension and flexion were measured using an isokinetic dynamometer at 60º/s, and the fear of falling was assessed using the *Falls Efficacy Scale–International* questionnaire. Both groups showed significant RTD_PEAK_ improvements from pre-intervention to the 12-month follow-up (*moderate*-to-*large* effect sizes). However, PA levels suggested to influence the retention effects: LAG showed declines in dominant RTD_PEAK_ (2% in extension and flexion) and in non-dominant side (6% extension and 5% flexion). In contrast, the MVAG maintained or slightly improved RTD_PEAK_ performance in dominant (1% extension and 2% flexion) and in non-dominant side (3% extension and 1% flexion). Fear of falling scores remained significantly reduced in both groups compared to pre-intervention, regardless of PA level. In summary, maintaining moderate-to-vigorous PA during follow-up appears to enhance the long-term retention of HSRT-induced neuromuscular adaptations, whereas HSRT effects on fear of falling were sustained regardless of PA level, highlighting its clinical potential for fall prevention.

**Supplementary Information:**

The online version contains supplementary material available at 10.1038/s41598-025-09095-8.

## Introduction

The global demographic shift toward an increasingly aged population, specifically individuals aged 65 and older, is occurring at an exponential rate, placing substantial demands on healthcare systems worldwide. Briefly, aging is an inevitable and universal biological process characterized by progressive declines in physiological functions, which increase vulnerability and, ultimately, lead to mortality^1^. Among these age-related impairments, the neuromuscular system is particularly affected^2^, primarily due to muscle mass atrophy, especially in type II (fast-twitch) fibers^3^ and a concurrent reduction in neuromuscular activation^4^. These factors collectively compromise the ability to produce force/torque rapidly, a key parameter known as the rate of torque development (RTD)^5^. Such declines negatively impact the daily lives of older adults by reducing functional capacity^6^ and increasing both the risk^7^ and fear of falls^8^.

In this context, structured exercise programs have been shown to effectively mitigate the detrimental effects of aging^9,10^. Among these, high-speed resistance training (HSRT) is widely recognized as a non-pharmacological and cost-effective intervention for older adults^10,12^ due to its ability to counteract neuromuscular deterioration associated with aging. Recent guidelines by Izquierdo et al.^10^ highlight the importance of HSRT interventions for older adults, emphasizing their ability to recruit fast-twitch muscle fibers, improve neuromuscular coordination through high-velocity contractions, optimize motor unit firing rates, and enhance both muscle activation and intermuscular coordination, factors closely related to RTD^13^.

Despite significant increases in RTD following resistance training (RT) programs in older adults^11,14,16^, some evidence suggests that these improvements are not sustained once structured and supervised RT is discontinued^15,17^. Unfortunately, as Douda et al.^18^ noted, the cessation or interruption of community-based exercise programs is often unavoidable. However, findings from Mertz et al.^19^ and Snijders et al.^20^ indicate that older adults who maintained RT participation at least once per week retained neuromuscular benefits, including RTD, muscle power, and quadriceps muscle cross-sectional area, over six-month and 12-month follow-up periods, respectively. These results underscore the importance of maintaining an *active lifestyle* and avoiding prolonged sedentary behaviors, such as excessive television viewing or mobile phone use, which are associated with reduced engagement in moderate-to-vigorous physical activity (PA) levels^21,23^.

Indeed, Izquierdo et al.^10^ emphasize that reduced PA habits can negatively impact muscle mass, power, strength, and functional capacity^24,25^. Consequently, discouraging older adults from maintaining or increasing their PA, or restricting their access to new exercise programs, raise important ethical concerns^9,10,26^. Rather than focusing solely on the traditional detraining effects, this study encouraged participants to sustain an *active lifestyle* following a 16-week HSRT program throughout a 12-month follow-up period. Thus, this study aimed to examine the role of PA levels in retaining RTD improvements over 12 months in independent older adults. Additionally, it explored whether PA levels contributed to sustaining the effects in fear of falling achieved during the intervention.

## Materials and methods

### Study design

This exploratory longitudinal study is part of the “*Idade Activa*” research project, registered *clinicaltrials.gov* (ID: NCT05586087 | https://www.clinicaltrials.gov/study/NCT05586087) with clinical trial registration on 19/10/2022. The project adhered to the principles outlined in the Declaration of Helsinki and was approved by the university’s Ethics Committee (approval no. 22030). All participants received detailed information regarding the study’s purpose, potential benefits, and associated risks, providing written informed consent prior to enrolment.

Participants underwent assessments at four distinct time points: baseline (M0), immediately after the intervention (M1), six months post-intervention (M2), and 12 months post-intervention (M3). The follow-up period consisted of two evaluations spaced six months apart.

For this analysis, only participants from the intervention group (IG) were included. Participants in the control group (CG) were placed on a waiting list at baseline (M0) to be enrolled in future research projects after the intervention, which excluded them from the current investigation.

The research committee overseeing the project recommended against the use of the term detraining, traditionally associated with a deliberate or imposed reduction PA over a defined timeframe. Instead, IG participants were encouraged to sustain their PA or engage in new exercise programs throughout the 12-month follow-up period.

### Participants

The present study focused exclusively on the participants who completed the 12-month follow-up assessment. As a result, the sample consisted of 36 older adults (mean age, 69.33 ± 3.12 years). After the follow-up, participants were categorized into two groups based on their PA, assessed by the *International Physical Activity Questionnaire–Short Form* (IPAQ-SF): the light activity group (LAG, *N* = 20, mean age, 70.00 ± 3.66 years) and the moderate-to-vigorous activity group (MVAG, *N* = 16, mean age, 68.50 ± 2.09 years). The general characteristics of the sample at baseline are presented in Table 1.

**Table 1:** General characteristics of the sample at baseline.

Measures	Groups	Baseline (M ± SD)	*p*-value
Age (years)	LAG	68.55 ± 3.52	0.221
	MVAG	67.31 ± 2.06	
Weight (kg)	LAG	67.89 ± 10.48	0.832
	MVAG	68.68 ± 11.39	
Height (m)	LAG	1.58 ± 0.07	0.291
	MVAG	1.56 ± 0.05	
BMI (kg/m^2^)	LAG	27.32 ± 4.32	0.490
	MVAG	28.26 ± 3.57	

*M* mean, *SD* standard deviation, *LAG* light activity group, *MVAG* moderate-to-vigorous activity group, *Kg* kilograms, *m* meters, *BMI* body mass index.

To qualify for inclusion in the study, participants needed to meet the following criteria: (a) be at least 65 years old; (b) walk independently without assistance; and (c) perform daily living activities autonomously. Exclusion criteria included having a diagnosis of diabetes or uncontrolled cardiac disease, undergoing surgery within the past six months, or living with an active oncological condition.

To enhance methodology transparency, we have included a supplementary Excel spreadsheet developed by Cheng^27^, which provides anonymized participant responses across the four assessment time points (M0, M1, M2, and M3). This file also contains the full set of standardized IPAQ-SF scoring criteria used to categorize participants into light, moderate, and vigorous PA levels, allowing readers to review and replicate the classification process in detail.

### Procedures

All assessments were conducted in the morning over two consecutive days. On the first day, anthropometric measurements were taken, while the second day was dedicated to assessing RTD and completing the *Falls Efficacy Scale–International* (FES-I) questionnaire. To ensure consistency, a single researcher carried out all measurements following the same sequence for each participant.

### Measurements

#### Rate of torque development

The RTD was measured by an isokinetic dynamometer (Biodex^®^ System 3, Biodex Corp., Shirley, New York, USA), which was configured and calibrated according to the manufacturer’s guidelines and a previous study^28^. Participants were seated with the lateral femoral epicondyle aligned with the lever arm’s rotation axis, maintaining both the knee and hip angles at 90º. Straps were used to secure participants around their hips and shoulders to maintain this position during muscle contractions, while the evaluated leg was firmly fixed to the lever arm. Initially, the participants were asked to relax to register the passive effect of gravity acting on the limb. Standardized verbal encouragement was provided during the assessment to motivate participants to exert maximal effort.

Before the measurement, the participants walked for 10 min at their fastest pace to warm. They then performed 10 repetitions at 210º/s to familiarize themselves with the equipment and minimize errors in subsequent repetitions. After the familiarization phase, three maximal isokinetic strength repetitions of the knee extensors (KE) and flexors (KF) were performed during concentric actions at 60º/s within a knee joint range of motion from 90º to 10º flexion (0º representing full KE) on both the dominant (DS) and non-dominant side (NDS). For RTD calculation, the torque–time curves from the three repetitions at 60º/s were analyzed.

The raw torque signal was measured in Nm, sampled at a rate of 100 Hz, and then subjected to a low-pass filter (Butterworth, 6 Hz, 2nd order). RTD is defined as the slope of the torque‒time curve^13^, during the following intervals after torque onset: 0–30 ms, 0–60 ms, 0–80 ms, 30–60 ms, 30–80 ms and 60–80 ms (RTD_0–30_, RTD_0–60_, RTD_0−80_, RTD_30–60_, RTD_30–80_, and RTD_60–80_). Torque onset was determined as the moment when the torque value reached 5 Nm. For statistical analysis, the trial yielding the highest RTD value within the 0 to 80 ms timeline was selected. Considering that repetition, all remaining RTD values for the time intervals were subsequently considered for analysis. RTD and its time intervals were analyzed via a custom routine developed with MathWorks software (Natick, Massachusetts, USA).

#### Falls efficacy scale-international

The FES-I questionnaire^29^ was used to evaluate participants’ concerns about falling during both basic and more demanding physical and social activities. This study employed the Portuguese version, translated from the Prevention of Falls Network Europe version^30^ and validated by Figueiredo and Santos^31^.

The questionnaire is divided into three sections based on activity type and includes 16 items. Each of them rated on a scale from one (not concerned at all) to four (very concerned). Scores from all items are summed up to produce a total score ranging from 16 to 64, with higher scores reflecting a greater fear of falling.

#### Physical activity

Participants’ PA levels were evaluated using the IPAQ-SF^32,33^, which collects information on multiple aspects of activity, including the frequency (days/week) and duration (minutes/week) of walking, moderate, and vigorous activities, as well as total PA expressed in MET-minutes/week. Additionally, sitting time during weekdays and weekends was recorded.

Based on their answers, which can be consulted in the supplementary Excel spreadsheet developed by Cheng^27^, participants were then categorized into light, moderate, or vigorous activity levels (as detailed in supplementary file as Table S1). In this file it is possible to note what are the criteria to divide the sample in these groups (the participants with moderate were merged with participants with high levels). In addition, participants were asked to report the main activities they had carried out in the last six months (i.e., from six- to the 12-month follow-up) [Table S2 in the supplementary file].

### Anthropometric

Anthropometric assessments included measuring weight and height by an electronic scale (TANITA^®^, MC 780MA, Amsterdam, Netherlands) and a stadiometer (SECA^®^ 220, Hamburg, Germany), respectively. Body mass index (BMI) was subsequently calculated via the standard formula: BMI = body mass (kg)/height^2^ (m^2^).

#### High-speed resistance training protocol

The HSRT program was performed three times weekly for 16 weeks under supervision. The prescription was adjusted every two weeks. Each session consisted of an initial warm-up (10 to 15-minute), the main HSRT exercises (45 to 55-minute), and a concluding cool-down phase (5 to 10-minute). The main phase included the following upper- and lower-body exercises: squats on smith machine or with dumbbells (depending on each participant’s ability); leg press, leg extension; calf raise; seated row; peck fly; lat pull down; and incline bench press (Technogym, SPA, Cesena, Italy).

This present training protocol employs progressively increasing loads, tailored to the participants’ mean concentric phase velocity for each set across all exercises^34,35^: 1st to 4th weeks, an average speed over 1.3 m/s was required (*starting strength*); from the 5th to 10th weeks, speeds were adjusted to between 1.3 and 1.0 m/s (*speed/strength*); and in the 11th to 16th weeks, speeds ranged from 1.0 to 0.75 m/s (*strength/speed*). The mean concentric phase velocity for each set and exercise was monitored using a BEAST™ sensor (Beast Technologies, Brescia, Italy)^36^. This device provided real-time feedback on instantaneous velocity to both participants and supervisors and displayed the mean velocity at the end of each set. Each session used six accelerometers connected via Bluetooth to six separate cell phones. Participants were also actively encouraged to execute each repetition swiftly and explosively, while maintaining a controlled pace of 2–3 s during the eccentric phase.

### Statistical analysis

Prior to the study, a sample size calculation was performed via G-power software (University of Dusseldorf, Germany)^37^ for *F* tests through ANOVA, repeated measured, within factors: f = 0.25, α error probability = 0.05, power (1-β err prob) = 0.80, number of groups = 1, and number of measurements = 4. The resulting power analysis indicated that this clinical trial should include at least 24 participants to achieve an 82% chance of successfully rejecting the null hypothesis. All statistical analyses were conducted using SPSS for Windows, version 26 (IBM Corp., Armonk, NY, USA), with the significance level set at *p* ≤ 0.050 (two-tailed). To complement traditional null hypothesis significance testing, an estimation-based analytical approach was employed^38,39^.

To evaluate the time effect across the four measurement points for each measure, repeated measures ANOVA were applied. Pairwise comparisons between time points and groups were conducted using the Bonferroni post-hoc test. The effect sizes (ESs) were then calculated according to Cohen^40^, using Cohen’s *d*_*unbiased*_ (*d*_*unb*_) through a specific spreadsheet^38^. While the ESs for ANOVA were expressed as partial eta-squared values (η_p_^2^) and interpreted using the following thresholds: 0.010–0.059 (small), 0.060–0.140 (medium), and greater than 0.140 (large), the pairwise comparisons’ ESs, expressed as *d*_*unb*_, were categorized as: less than 0.20 (trivial), 0.20–0.49 (small), 0.50–0.80 (medium), and greater than 0.80 (large). Graphical data representations were generated created using RStudio software.

## Results

### Participants

The participants’ general characteristics at all time points of the study are presented in Table 2. Several significant differences were observed in age, weight, and BMI over the study period.

**Table 2:** General characteristics of the sample at all time moments (Mean ± SD).

Measures	Groups	Intervention		Follow-up		Interaction effect Within groups	Interaction effect Between groups
		M0 Pre	M1 Post	M2 6-Months	M3 12-Months		
Age (years)	LAG ^a, b,c^	68.55 ± 3.52	69.15 ± 3.57	69.45 ± 3.52	70.00 ± 3.66	F = 2.756¥ *p* = 0.068 η²_p_=0.075*	F = 0.013 *p* = 0.908 η²_p_=0.001
	MVAG ^b, c,d, e^	67.31 ± 2.06	67.50 ± 2.09	68.19 ± 2.01	68.50 ± 2.09		
Weight (kg)	LAG	67.89 ± 10.48	67.93 ± 11.37	66.64 ± 11.51	66.59 ± 11.01	F = 2.522¥ *p* = 0.086 η²_p_=0.069*	F = 0.110 *p* = 0.742 η²_p_=0.003
	MVAG ^b, c,e^	68.68 ± 11.39	67.52 ± 11.90	66.32 ± 12.00	64.79 ± 11.89		
BMI (kg/m^2^)	LAG	27.32 ± 4.32	27.31 ± 4.51	26.75 ± 4.47	26.68 ± 4.24	F = 1.650 *p* = 0.182 η²_p_=0.046#	F = 0.217 *p* = 0.644 η²_p_=0.006
	MVAG ^b, c,e^	28.26 ± 3.57	27.76 ± 3.78	27.26 ± 3.88	26.58 ± 3.68		

*M* mean, *SD* standard deviation, *LAG* light activity group, *MVAG* moderate-to-vigorous activity group, *Kg* kilograms, *BMI* body mass index, *m* meters.

^¥^Greenhouse-Geisser correction.

Significant differences:.

a, pre-intervention vs. post-intervention; b, pre-intervention vs. 6-month follow-up; c, pre-intervention vs. 12-month follow-up; d, post-intervention vs. 6-month follow-up;.

e, post-intervention vs. 12-month follow-up; f, 6-month follow-up vs. 12-month follow-up.

η²_p_ values thresholds:.

#, small effect: 0.010 to 0.059; *, medium effect: 0.060 to 0.140; §, large effect: > 0.140.

### Rate of torque development

Table 3 shows the changes in RTD values over the study period for KE. Several RTD values and their time intervals demonstrated medium to large time effect throughout the study period.

**Table 3:** Changes in rate of torque development for knee extension over the study period.

Measures	Groups	Intervention		Follow-up		Time Effect	Interaction effect Within groups	Interaction effect Between groups
		M0 Pre	M1 Post	M2 6-Month	M3 12-Month			
*Extension | Dominant Side*								
RTD_PEAK_ (Nm/sec)	LAG ^a, b,c^	197.67 ± 56.96	261.81 ± 71.73	262.18 ± 79.32	256.12 ± 87.04	F = 19.485¥ ***p*** **< 0.001** η²_p_=0.364§	F = 0.147¥ *p* = 0.886 η²_p_=0.004	F = 1.332 *p* = 0.257 η²_p_=0.038#
	MVAG ^a, b,c^	221.33 ± 98.82	292.45 ± 106.29	289.95 ± 92.09	293.49 ± 99.72			
RTD_0–30_ (Nm/sec)	LAG ^a, b^	192.23 ± 58.56	261.81 ± 71.73	262.18 ± 79.32	245.61 ± 85.41	F = 14.796 ***p*** **< 0.001** η²_p_=0.303§	F = 0.623 *p* = 0.602 η²_p_=0.018#	F = 1.390 *p* = 0.247 η²_p_=0.039#
	MVAG ^a, b,c^	221.33 ± 98.82	292.45 ± 106.29	277.15 ± 114.16	293.49 ± 99.72			
RTD_0–60_ (Nm/sec)	LAG ^a, e^	125.46 ± 47.77	149.16 ± 45.65	127.37 ± 42.77	133.10 ± 48.53	F = 4.330¥ ***p*** **= 0.013** η²_p_=0.113*	F = 0.735¥ *p* = 0.498 η²_p_=0.021#	F = 0.390 *p* = 0.582 η²_p_=0.009
	MVAG	133.41 ± 47.82	147.61 ± 44.00	138.00 ± 56.69	148.06 ± 49.19			
RTD_0−80_ (Nm/sec)	LAG	86.28 ± 35.56	93.55 ± 32.56	76.80 ± 29.47	83.81 ± 34.77	F = 5.239¥ ***p*** **= 0.004** η²_p_=0.134*	F = 0.116¥ *p* = 0.922 η²_p_=0.003	F = 0.007 *p* = 0.935 η²_p_=0.001
	MVAG	86.75 ± 25.13	95.41 ± 33.24	73.52 ± 40.96	81.48 ± 43.63			
RTD_30–60_ (Nm/sec)	LAG ^b, d^	60.39 ± 69.95	38.88 ± 41.44	−4.62 ± 48.79	23.45 ± 92.83	F = 9.243 ***p*** **< 0.001** η²_p_=0.214§	F = 0.662 *p* = 0.577 η²_p_=0.019#	F = 0.784 *p* = 0.382 η²_p_=0.023#
	MVAG	47.72 ± 30.99	15.45 ± 50.47	1.09 ± 42.30	5.80 ± 41.72			
RTD_30–80_ (Nm/sec)	LAG ^**b**^	24.44 ± 48.15	−5.09 ± 36.68	−29.04 ± 40.17	−10.87 ± 65.15	F = 10.333 ***p*** **< 0.001** η²_p_=0.233§	F = 0.283 *p* = 0.838 η²_p_=0.008	F = 2.661 *p* = 0.112 η²_p_=0.073*
	MVAG ^b, c^	8.13 ± 36.72	−21.10 ± 50.73	−46.36 ± 75.01	−42.85 ± 61.43			
RTD_60–80_ (Nm/sec)	LAG	−29.53 ± 43.07	−71.06 ± 59.79	−76.14 ± 58.93	−62.87 ± 52.49	F = 5.180¥ ***p*** **= 0.006** η²_p_=0.132*	F = 0.896¥ *p* = 0.423 η²_p_=0.026#	F = 2.703 *p* = 0.109 η²_p_=0.074*
	MVAG	−51.06 ± 67.29	−76.36 ± 63.93	−116.91 ± 145.41	−115.03 ± 105.85			
*Extension | Non-dominant Side*								
RTD_PEAK_ (Nm/sec)	LAG ^a, b,c^	211.42 ± 59.59	295.53 ± 75.33	276.59 ± 83.16	276.79 ± 75.55	F = 28.996 ***p*** **< 0.001** η²_p_=0.460§	F = 1.798 *p* = 0.152 η²_p_=0.050#	F = 0.001 *p* = 0.995 η²_p_=0.001
	MVAG ^a, b,c^	225.88 ± 108.80	281.66 ± 92.12	263.19 ± 92.05	290.23 ± 94.22			
RTD_0–30_ (Nm/sec)	LAG ^a, b,c^	211.17 ± 60.08	295.53 ± 75.33	276.59 ± 83.16	276.78 ± 75.55	F = 29.059 ***p*** **< 0.001** η²_p_=0.461§	F = 1.812 *p* = 0.150 η²_p_=0.051#	F = 0.001 *p* = 0.993 η²_p_=0.001
	MVAG ^a, b,c^	225.88 ± 108.80	281.66 ± 92.12	263.19 ± 92.04	290.23 ± 94.22			
RTD_0–60_ (Nm/sec)	LAG ^a^	132.93 ± 26.88	156.36 ± 30.52	141.56 ± 36.61	142.96 ± 45.90	F = 12.065 ***p*** **< 0.001** η²_p_=0.262§	F = 0.390 *p* = 0.761 η²_p_=0.011#	F = 0.039 *p* = 0.844 η²_p_=0.001
	MVAG ^a, d^	128.63 ± 41.38	158.06 ± 54.86	134.32 ± 44.61	142.70 ± 48.65			
RTD_0−80_ (Nm/sec)	LAG	91.43 ± 23.71	91.52 ± 32.43	79.45 ± 33.77	83.92 ± 31.46	F = 5.429¥ ***p*** **= 0.004** η²_p_=0.138*	F = 0.807¥ *p* = 0.471 η²_p_=0.023#	F = 0.575 *p* = 0.454 η²_p_=0.017#
	MVAG ^c, e^	89.68 ± 34.16	91.19 ± 49.26	67.44 ± 35.47	69.41 ± 37.45			
RTD_30–60_ (Nm/sec)	LAG ^a, b,c^	56.68 ± 35.53	20.79 ± 46.68	10.76 ± 46.08	12.15 ± 37.82	F = 7.940 ***p*** **< 0.001** η²_p_=0.189§	F = 1.673 *p* = 0.177 η²_p_=0.047#	F = 0.559 *p* = 0.460 η²_p_=0.016#
	MVAG	33.23 ± 35.62	36.77 ± 43.42	7.67 ± 38.22	−2.12 ± 49.78			
RTD_30–80_ (Nm/sec)	LAG ^a, b,c^	21.48 ± 27.75	−27.76 ± 50.31	−35.68 ± 60.03	−29.17 ± 30.62	F = 14.079 ***p*** **< 0.001** η²_p_=0.293§	F = 1.033 *p* = 0.381 η²_p_=0.029#	F = 0.993 *p* = 0.326 η²_p_=0.028#
	MVAG **b, c**	9.55 ± 17.11	−20.25 ± 60.55	−47.58 ± 77.72	−57.99 ± 57.50			
RTD_60–80_ (Nm/sec)	LAG ^a^	−30.89 ± 52.32	−101.19 ± 72.67	−105.34 ± 99.43	−91.71 ± 49.39	F = 10.529¥ ***p <*** **0.001** η²_p_=0.236*	F = 1.034¥ *p* = 0.370 η²_p_=0.030#	F = 0.772 *p* = 0.386 η²_p_=0.022#
	MVAG ^a, b,c^	−26.02 ± 33.97	−104.04 ± 112.82	−129.09 ± 163.51	−147.08 ± 121.61			

Significant differences between periods are highlighted in bold (*p* ≤ 0.05). Abbreviations: LAG, light activity group; MVAG, moderate-to-vigorous activity group; RTD, rate of torque development; Nm, newton meter; sec, seconds.

¥, Greenhouse‒Geisser correction.

Significant differences:.

a, pre-intervention vs. post-intervention; b, pre-intervention vs. 6-month follow-up; c, pre-intervention vs. 12-month follow-up; d, post-intervention vs. 6-month follow-up;.

e, post-intervention vs. 12-month follow-up;.

η²_p_ values thresholds:.

#, small effect: 0.010 to 0.059; *, medium effect: 0.060 to 0.140; §, large effect: > 0.140.

Table 3 also presents the interaction effects both within and between groups. Although no significant between-group differences were observed, several significant within-group changes were identified over time.

First, for RTD_PEAK_ during KE-DS, both groups demonstrated significantly lower values at baseline compared to post-intervention (LAG: *p* = 0.002, *d*_*unb*_=0.95 [0.45 to 1.52]; MVAG: *p* = 0.003, *d*_*unb*_=0.66 [0.22 to 1.15]), six-month (LAG: *p* = 0.001, *d*_*unb*_=0.89 [0.48 to 1.37]; MVAG: *p* = 0.001, *d*_*unb*_=0.68 [0.24 to 1.18]), and 12-month follow-up (LAG: *p* = 0.018, *d*_*unb*_=0.76 [0.28 to 1.29]; MVAG: *p* = 0.007, *d*_*unb*_=0.69 [0.19 to 1.24]). Moreover, during KE-NDS, both groups exhibited significantly lower values at baseline compared to post-intervention (LAG: *p* < 0.001, *d*_*unb*_=1.19 [0.70 to 1.76]; MVAG: *p* = 0.001, *d*_*unb*_=0.53 [0.26 to 0.84]), six-month (LAG: *p* < 0.001, *d*_*unb*_=0.87 [0.46 to 1.33]; MVAG: *p* = 0.030, *d*_*unb*_=0.35 [0.12 to 0.62]), and 12-month follow-ups (LAG: *p* < 0.001, *d*_*unb*_=0.92 [0.45 to 1.45]; MVAG: *p* = 0.001, *d*_*unb*_=0.60 [0.27 to 0.98]).

**Table 4:** Changes in rate of torque development for knee flexion over the study period.

Measures	Groups	Intervention		Follow-up		Time Effect	Interaction effect Within groups	Interaction effect Between groups
		M0 Pre	M1 Post	M2 6-Month	M3 12-Month			
*Flexion | Dominant Side*								
RTD_PEAK_ (Nm/sec)	LAG ^a, b,c^	118.89 ± 63.05	159.11 ± 89.35	171.48 ± 95.09	155.83 ± 79.43	F = 17.456¥ ***p*** **< 0.001** η²_p_=0.339§	F = 0.070¥ *p* = 0.950 η²_p_=0.002	F = 0.006 *p* = 0.939 η²_p_=0.001
	MVAG ^a, b,c^	113.96 ± 49.15	155.59 ± 54.72	171.33 ± 74.67	157.43 ± 63.60			
RTD_0–30_ (Nm/sec)	LAG ^b, c^	98.43 ± 27.51	130.63 ± 30.64	140.89 ± 31.15	133.96 ± 40.59	F = 10.689¥ ***p*** **< 0.001** η²_p_=0.239§	F = 0.418¥ *p* = 0.666 η²_p_=0.012#	F = 0.213 *p* = 0.648 η²_p_=0.006
	MVAG ^b, c^	99.77 ± 69.62	128.09 ± 96.62	152.56 ± 56.11	149.34 ± 57.78			
RTD_0–60_ (Nm/sec)	LAG ^b^	63.72 ± 14.74	75.80 ± 15.31	80.19 ± 17.80	77.58 ± 21.36	F = 8.706¥ ***p*** **< 0.001** η²_p_=0.204§	F = 0.847¥ *p* = 0.444 η²_p_=0.024#	F = 0.270 *p* = 0.607 η²_p_=0.008
	MVAG ^b, c^	62.53 ± 37.77	75.06 ± 48.06	87.94 ± 30.65	88.29 ± 38.05			
RTD_0−80_ (Nm/sec)	LAG ^b^	51.98 ± 13.09	64.23 ± 18.94	69.31 ± 17.23	64.41 ± 17.15	F = 11.083 ***p*** **< 0.001** η²_p_=0.246§	F = 0.852 *p* = 0.469 η²_p_=0.024#	F = 1.271 *p* = 0.267 η²_p_=0.036#
	MVAG ^b, c^	53.63 ± 31.13	70.38 ± 37.09	83.54 ± 36.58	75.54 ± 37.75			
RTD_30–60_ (Nm/sec)	LAG	29.72 ± 21.95	21.70 ± 15.09	19.56 ± 8.46	23.95 ± 16.99	F = 1.552¥ *p* = 0.214 η²_p_=0.044#	F = 0.860¥ *p* = 0.445 η²_p_=0.025#	F = 0.855 *p* = 0.362 η²_p_=0.025#
	MVAG	26.27 ± 18.88	22.64 ± 10.02	23.52 ± 16.22	34.45 ± 34.32			
RTD_30–80_ (Nm/sec)	LAG	24.64 ± 17.04	24.77 ± 18.74	26.27 ± 12.99	25.49 ± 15.21	F = 1.814¥ *p* = 0.161 η²_p_=0.051#	F = 1.272¥ *p* = 0.288 η²_p_=0.036#	F = 2.978 *p* = 0.093 η²_p_=0.081*
	MVAG	26.54 ± 17.43	35.86 ± 29.70	41.88 ± 38.69	40.63 ± 33.93			
RTD_60–80_ (Nm/sec)	LAG	16.73 ± 13.82	28.97 ± 40.05	36.03 ± 29.61	27.74 ± 27.65	F = 3.655¥ ***p*** **= 0.034** η²_p_=0.097*	F = 0.396¥ *p* = 0.661 η²_p_=0.012#	F = 3.420 *p* = 0.073 η²_p_=0.091*
	MVAG	30.53 ± 29.02	54.69 ± 71.03	68.36 ± 93.99	54.55 ± 67.43			
*Flexion | Non-dominant Side*								
RTD_PEAK_ (Nm/sec)	LAG ^a, b,c^	102.65 ± 25.33	151.66 ± 45.52	142.03 ± 31.57	143.27 ± 31.37	F = 13.464 ***p*** **< 0.001** η²_p_=0.284§	F = 0.381 *p* = 0.767 η²_p_=0.011#	F = 2.491 *p* = 0.124 η²_p_=0.068*
	MVAG ^a, b,c^	124.95 ± 56.15	162.57 ± 48.89	169.81 ± 81.56	163.43 ± 54.86			
RTD_0–30_ (Nm/sec)	LAG ^a, c^	101.29 ± 27.98	140.10 ± 30.42	141.41 ± 31.84	137.24 ± 28.47	F = 6.955¥ ***p*** **= 0.002** η²_p_=0.170§	F = 1.489¥ *p* = 0.233 η²_p_=0.042#	F = 1.726 *p* = 0.198 η²_p_=0.048#
	MVAG ^a, c^	124.96 ± 56.15	162.56 ± 48.89	132.72 ± 98.81	163.38 ± 54.91			
RTD_0–60_ (Nm/sec)	LAG ^a, c^	63.84 ± 11.74	80.46 ± 21.44	85.02 ± 23.68	82.34 ± 15.73	F = 5.587¥ ***p*** **= 0.008** η²_p_=0.141§	F = 1.434¥ *p* = 0.246 η²_p_=0.040#	F = 0.825 *p* = 0.370 η²_p_=0.024#
	MVAG ^a, c^	74.73 ± 30.95	89.77 ± 26.44	78.95 ± 53.03	94.01 ± 28.11			
RTD_0−80_ (Nm/sec)	LAG ^a, c^	51.83 ± 8.16	71.68 ± 25.29	64.35 ± 31.10	69.15 ± 16.18	F = 4.523¥ ***p*** **= 0.016** η²_p_=0.117*	F = 0.228¥ *p* = 0.782 η²_p_=0.007	F = 1.085 *p* = 0.305 η²_p_=0.031#
	MVAG ^c^	62.18 ± 29.19	74.52 ± 22.48	73.84 ± 50.59	76.07 ± 26.75			
RTD_30–60_ (Nm/sec)	LAG	27.44 ± 13.99	21.58 ± 14.68	22.10 ± 14.76	24.77 ± 11.65	F = 0.729 *p* = 0.537 η²_p_=0.021#	F = 0.349 *p* = 0.790 η²_p_=0.010#	F = 0.035 *p* = 0.852 η²_p_=0.001
	MVAG	25.37 ± 14.57	25.36 ± 11.64	22.65 ± 14.18	24.91 ± 17.74			
RTD_30–80_ (Nm/sec)	LAG	22.88 ± 12.92	30.89 ± 31.01	27.49 ± 19.94	26.66 ± 17.59	F = 2.071¥ *p* = 0.133 η²_p_=0.057#	F = 1.852¥ *p* = 0.164 η²_p_=0.052#	F = 0.899 *p* = 0.350 η²_p_=0.026#
	MVAG	25.04 ± 16.18	25.35 ± 10.96	43.21 ± 45.70	35.11 ± 17.33			
RTD_60–80_ (Nm/sec)	LAG	15.95 ± 17.04	44.45 ± 71.09	31.94 ± 34.09	29.30 ± 39.59	F = 2.282¥ *p* = 0.115 η²_p_=0.063*	F = 1.925¥ *p* = 0.158 η²_p_=0.054#	F = 0.511 *p* = 0.480 η²_p_=0.015#
	MVAG	24.25 ± 26.97	25.52 ± 17.53	63.85 ± 106.86	40.34 ± 35.88			

Significant differences between periods are highlighted in bold (*p* ≤ 0.05). Abbreviations: LAG, light activity group; MVAG, moderate-to-vigorous activity group; RTD, rate of torque development; Nm, newton meter; sec, seconds.

¥, Greenhouse‒Geisser correction.

Significant differences:.

a, pre-intervention vs. post-intervention; b, pre-intervention vs. 6-month follow-up; c, pre-intervention vs. 12-month follow-up; d, post-intervention vs. 6-month follow-up;.

e, post-intervention vs. 12-month follow-up;.

η²_p_ values thresholds:.

#, small effect: 0.010 to 0.059; *, medium effect: 0.060 to 0.140; §, large effect: > 0.140.

Table 4 shows the changes in RTD values over the study period for KF. Several RTD values and their time intervals demonstrated medium to large time effect throughout the study period.

**Fig. 1:**
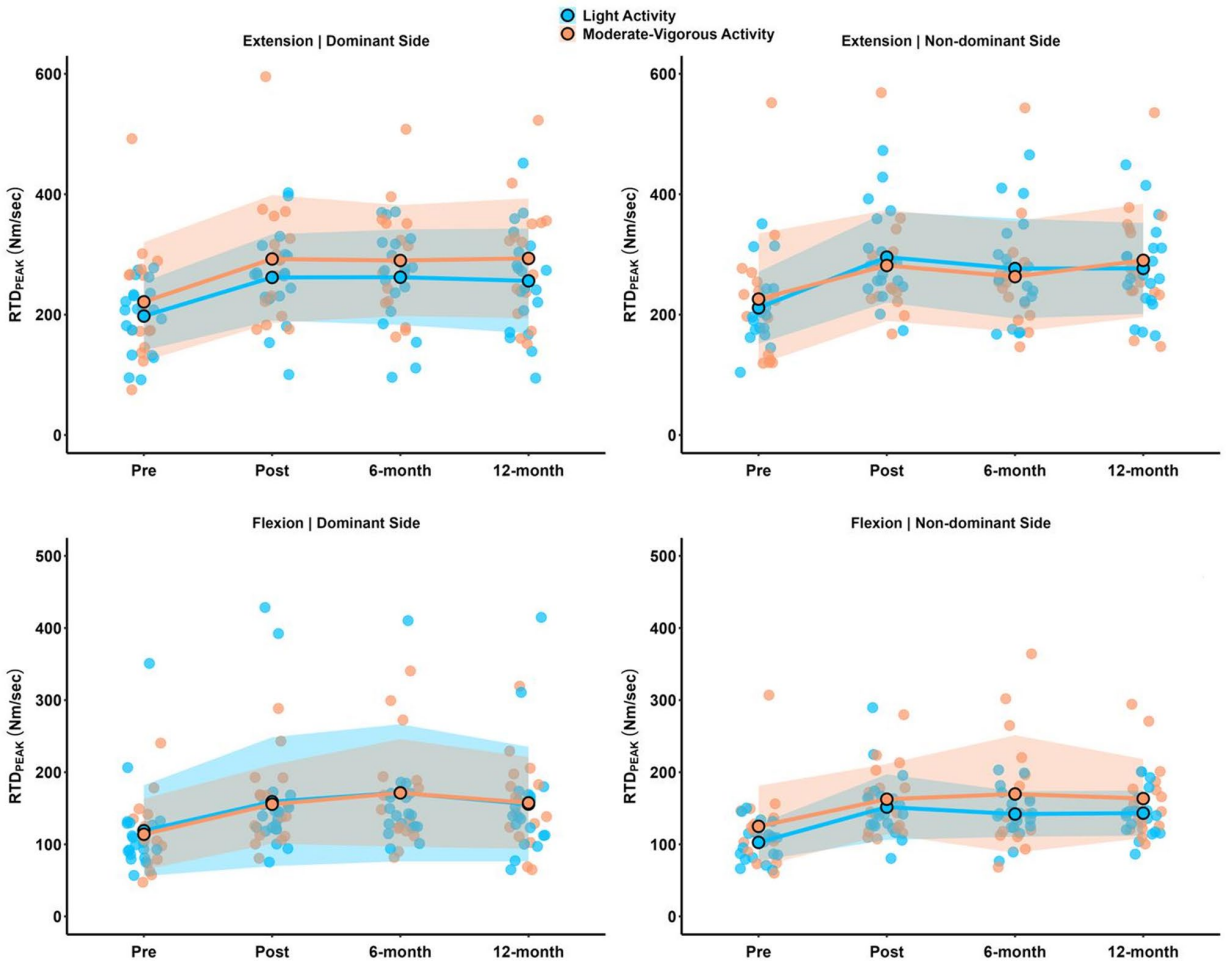
Rate of torque values for light activity and moderate-to-vigorous activity groups. Solid lines and filled dots represent mean values, while shaded areas indicate the standard deviation. *RTD*, rate of torque development.

For RTD_PEAK_ during KF-DS, both groups revealed significantly lower values at baseline compared to post-intervention (LAG: *p* = 0.006, *d*_*unb*_=0.49 [0.22 to 0.81]; MVAG: *p* = 0.013, *d*_*unb*_=0.76 [0.17 to 1.41]), six-month (LAG: *p* = 0.002, *d*_*unb*_=0.63 [0.31 to 0.98]; MVAG: *p* = 0.003, *d*_*unb*_=0.86 [0.26 to 1.54]), and 12-month follow-ups (LAG: *p* = 0.031, *d*_*unb*_=0.49 [0.13 to 0.89]; MVAG: *p* = 0.021, *d*_*unb*_=0.73 [0.22 to 1.29]). Lastly, during KF-NDS, both groups revealed significantly lower values at baseline than post-intervention (LAG: *p* = 0.001, *d*_*unb*_=1.28 [0.55 to 2.08]; MVAG: *p* = 0.031, *d*_*unb*_=0.68 [0.21 to 1.20]), six-month (LAG: *p* = 0.017, *d*_*unb*_=1.32 [0.65 to 2.08]; MVAG: *p* = 0.014, *d*_*unb*_=0.61 [0.09 to 1.18]), and 12-month follow-ups (LAG: *p* = 0.004, *d*_*unb*_=1.37 [0.74 to 2.09]; MVAG: *p* = 0.017, *d*_*unb*_=0.66 [0.09 to 1.28]). For clarity, we report here only the results for KE and KF for RTD_PEAK_. The results for the RTD time intervals, as indicated in Tables 3 and 4, are provided in the supplementary file. Figure 1 illustrates the changes in KE and KF for both sides of RTD_PEAK_.

### Falls efficacy scale-international

Table 5 presents the changes in fear of falling across the four measurement points.

**Table 5:** Changes in fear of falling score over the study period.

Measure	Groups	Intervention		Follow-up		Time Effect	Interaction effect Within groups	Interaction effect Between groups
		M0 Pre	M1 Post	M2 6-Month	M3 12-Month			
FFS-I (score)	LAG	22.90 ± 4.98	20.80 ± 5.19	19.10 ± 1.94	19.60 ± 2.72	F = 14.360¥ ***p*** **< 0.001** η²_p_=0.297§	F = 0.201¥ *p* = 0.839 η²_p_=0.006	F = 0.079 *p* = 0.781 η²_p_=0.002
	MVAG	23.75 ± 6.13	21.31 ± 5.71	19.19 ± 3.29	19.44 ± 2.48			

Significant differences between periods are highlighted in bold (*p* ≤ 0.05). Abbreviations: LAG, light activity group; MVAG, moderate-to-vigorous activity group; FFS-I, falls efficacy scale-international.

¥, Greenhouse‒Geisser correction.

η²_p_ values thresholds:.

#, small effect: 0.010 to 0.059; *, medium effect: 0.060 to 0.140; §, large effect: > 0.140.

**Fig. 2:**
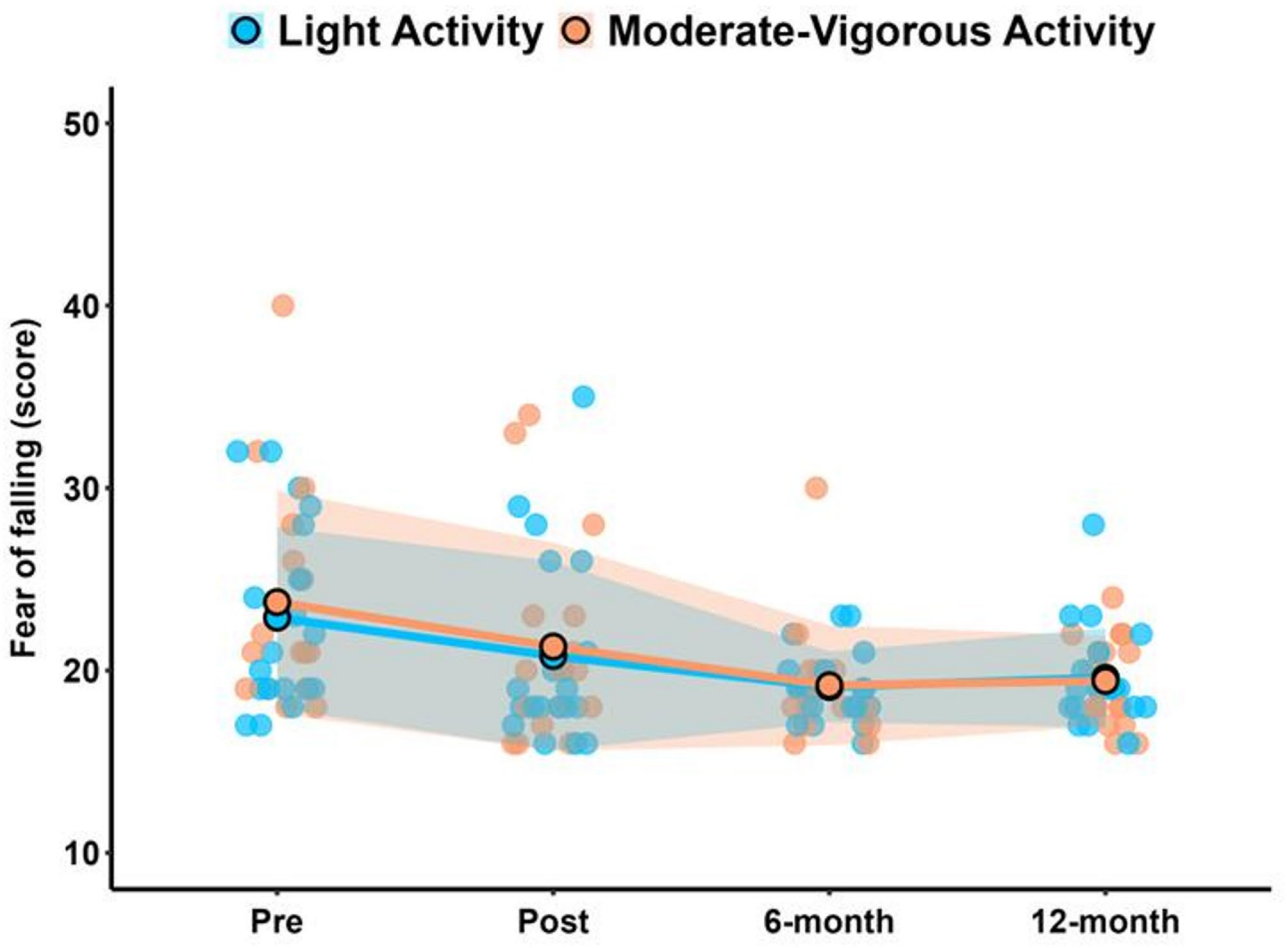
Fear of falling values for light activity and moderate-to-vigorous activity groups. Solid lines and filled dots represent mean values, while shaded areas indicate the standard deviation.

Although a significant time effect was observed during the study period, no significant differences were identified either between or within the groups. Figure 2 illustrates the fear of falling pattern over the study period for both groups.

## Discussion

This exploratory study examined whether different PA levels influenced the long-term retention of improvements in RTD achieved through a 16-week HSRT program in independent older adults, assessed over a 12-month follow-up period. Additionally, changes in fear of falling over time were analyzed. The key findings were as follows: (i) regardless of PA levels, the RTD_PEAK_ values for KE and KF remained significantly higher at the 12-month follow-up than baseline [*small* to *large* ESs] (Fig. 1); (ii) except for RTD_PEAK_ values for KF-DS, the MVAG participants showed higher values at the 12-month follow-up compared to the LAG [*small* ESs]; (iii) the MVAG also exhibited a greater ability to generate force in the early phases of contraction (i.e., RTD_0–30_, RTD_0–60_) at the 12-month follow-up compared to the LAG [*trivial* to *medium* ESs]; and (iv) both groups demonstrated an improvement in their fear of falling values at the 12-month follow-up compared to baseline [LAG: *medium* ES; MVAG: *large* ES]. These findings are clinically meaningful, as older adults with reduced muscle power often struggle to rapidly generate sufficient torque or force to restore postural control in response to perturbations^41^. This underscores the importance of prioritizing muscle power, particularly RTD, in interventions within this population.

It is important to highlight that, unlike previous studies examining detraining effects on RTD in older adults^11,15,17^, which associated these effects with the absence of exercise stimuli, this study focused on sustaining lifestyle habits to address ethical concerns^26^, encouraging participants to maintain or increase their PA levels post-intervention. As detailed in Supplementary Table S2, ten participants started new exercise programs during the follow-up period.

This study presents clinically meaningful and sustained improvements in RTD_PEAK_ for both groups, sustained from baseline to the 12-month follow-up. Specifically, KE-DS improved by 29% and 32% for the LAG and MVAG, respectively, while KF-DS improved by 31% and 38%. Similarly, substantial increases were noted for the NDS. While KE enhanced by 30% and 28% for the LAG and MVAG, respectively, the KF increased 39% and 30%, respectively. These improvements align with the thresholds established by Kirn et al.^42^, who defined clinically meaningful leg-extensor power effects as 9% to 10%, and substantial improvements as 15% to 18% in mobility-limited older adults. While this study focused on independent older adults, the findings suggest that HSRT could be an effective strategy to mitigate and prevent age-related neuromuscular decline.

Additionally, previous research suggests that RT programs lasting more than 12 weeks, particularly those incorporating explosive movements and controlled velocity, may minimize losses in neuromuscular function following intervention cessation^11,14,16^. In this study, the use of accelerometers to ensure adherence to specific velocity zones during HSRT may have contributed to reverse (or delay) the decline in the ability to rapidly develop force in these participants. Future studies should compare the long-term effects of traditional RT and HSRT to elucidate the potential role of contraction velocity in preserving neuromuscular function.

Nevertheless, PA levels played a critical role in preserving RTD_PEAK_ effects. Over the study period (M0 vs. M3), participants who substantially reduced their PA after post-intervention experienced declines of 2% in both KE-DS and KF-DS, with greater reductions on the NDS, 6% and 5%, respectively. In contrast, the MVAG maintained their performance. These findings align with the results of Mertz et al.^19^, who reported that participants who continued RT at least once per week presented a better preservation in force development values [*small* ES] over six months. Moreover, the MVAG’s superior ability to generate torque within shorter time intervals (in the supplementary file as Fig. S1 to S4), suggests a more economical neuromuscular system^43^, which is critical for fall prevention^44^.

On the other hand, the previous studies which have focused on detraining effects have reported inconsistent results in RTD values following RT interventions. While Sakugawa et al.^17^ and Lovell et al.^15^ observed decreases over four-month and one-month follow-ups, respectively, Baxter et al.^11^ found greater retention of training-induced improvements over three months. In addition, the 12-month follow-up data for LAG suggest a more pronounced decline compared to Reid et al.^45^, who reported significant three-year reductions of 9% and 8% in muscle power, and 8% and 13% in contraction velocity, in healthy and mobility-limited older adults. These findings are practically significant, as potential losses in force production can lead to reductions in functional capacity^6^, and, ultimately, an increased risk of falls^7^. Participants who discontinued at least moderate PA and RT at least once weekly may experience neuromuscular impairments, including selective atrophy of the largest and fastest-contracting muscle fibers^46^, increased antagonist muscle co-contraction^47^, and accelerated demyelination impairing neural transmission of motor commands^48^.

In addition to these results, the fear of falling was assessed as a secondary aim using the FES-I, which is the most widely used questionnaire in this population^49^. The results demonstrated a significant main benefit time effect [*large* ES] across the study period for all participants, irrespective of PA levels (Table 5). Within-group analyses also revealed improvements in both groups from baseline to the 12-month follow-up [LAG: *medium* ES; MVAG: *large* ES]. These improvements may be related to the observed enhancements in neuromuscular function (M0 vs. M3). Indeed, recent systematic review findings^49^ suggest that a reduced concern about falling may be related to improvements in motor function. Moreover, the sustained benefits observed during the follow-up period provide valuable insights beyond a previous systematic review^50^, which highlighted uncertainty regarding the sustainability of intervention effects after cessation. The *medium*-to-*large* ESs observed in this study are clinically relevant, as fear of falling is significantly associated with the occurrence of falls in the previous month or year^51^. These findings underscore the importance of addressing fear of falling in future research.

Despite the insights provided, the present study presents some limitations that warrant consideration. First, although a priori power analysis indicated that a sample of 24 participants would be sufficient to detect meaningful effects with 82% power, the final sample sizes in subgroup analyses may limit the generalizability of the findings. Second, PA levels were assessed using the IPAQ-SF, a validated tool^32,52^, but it relies on self-reported and may be subject to recall bias and overestimation, namely among older adults. While objective tools such as accelerometers could improve measurement accuracy, their higher cost may limit feasibility. Third, the assessor was aware of the study’s objectives, which may have introduced bias through expectancy effects. Lastly, caloric intake was not controlled during the follow-up period, which could have influenced the outcomes.

## Conclusions

The findings of this study emphasize that the improvements in RTD and fear of falling scores achieved through the 16-week HSRT program were sustained over a 12-month follow-up period. Importantly, the results also suggest that maintaining moderate-to-vigorous PA levels post-intervention further enhanced the retention of neuromuscular benefits and contributed to a more efficient neuromuscular system, which is critical for fall prevention. In addition, the reduction in fear of falling observed across all participants, regardless of PA levels, suggests a link to the improvements in neuromuscular function.

### Implications for practice

Given the importance of maintaining RTD in older adults to effectively respond to perturbations and reduce the risk of falls, and considering that the RTD improvements observed in this study exceeded established thresholds for clinically meaningful changes^42^, it is essential to implement health policies that promote active aging and neuromuscular health. These policies should also encourage collaboration between healthcare and exercise professionals to conduct longitudinal assessments of key health parameters, such as RTD, in clinical and community health settings. Additionally, practitioners should be aware of the potential influence of PA levels on long-term retention of training effects.

For future research, we recommend comparing traditional RT and HSRT programs to better understand the role of contraction velocity in preserving neuromuscular function in older adults, with consideration of PA levels during the follow-up period. Importantly, future studies should also examine the relationship between RTD and functional outcomes, such as balance and gait performance, to enhance clinical applicability. Finally, evaluating the magnitude and sustainability of RTD adaptations after exercise cessation, followed by the reintroduction of HSRT, could offer valuable insights into optimal program design for older adults.

## Electronic supplementary material

Below is the link to the electronic supplementary material.

Supplementary Material 1

Supplementary Material 2

## Data Availability

Data available on request from the corresponding author.
