# Supplementary material for: Long-term effects on rate of torque development and fear of falling following high-speed resistance training in older adults
Source: Sci Rep. 2025 Aug 9;15:29139. doi: 10.1038/s41598-025-09095-8 (PMC12335496; doi:10.1038/s41598-025-09095-8)
Supplement: Supplementary file 2 — Supplementary Material 2 [file 41598_2025_9095_MOESM2_ESM.docx]

**Full description of the RTD time intervals results**

Knee Extension (KE) for dominant side (DS)

For RTD_0-30_, both groups demonstrated significantly lower values at baseline than post-intervention (LAG: *p*=0.001, *d_unb_*=1.02 [0.50 to 1.61]; MVAG: *p*=0.003, *d_unb_*=0.66 [0.22 to 1.15]), and six-month follow-up (LAG: *p*=0.001, *d_unb_*=0.96 [0.48 to 1.51]; MVAG: *p*=0.037, *d_unb_*=0.49 [0.07 to 0.96]). Additionally, MVAG values were significantly higher at the 12-month follow-up compared to baseline (*p*=0.014, *d_unb_*=0.69 [0.19 to 1.24]). For RTD_0-60_, LAG revealed significantly higher values at post- than baseline (*p*=0.003, *d_unb_*=0.49 [0.19 to 0.81]), and 12-month follow-up (*p*=0.027, *d_unb_*=-0.33 [-0.56 to -0.11]). For RTD_30–60_, LAG exhibited significantly lower values at six-month follow-up than baseline (*p*=0.001, *d_unb_*=-1.04 [-1.75 to -0.39]), and post-intervention (*p*=0.017, *d_unb_*=-0.92 [-1.64 to -0.27]). For RTD_30–80_, both groups presented significantly lower values at six-month follow-up than baseline (LAG: *p*=0.017, *d_unb_*=-1.16 [-1.93 to -0.45]; MVAG: *p*=0.037, *d_unb_*=-0.88 [-1.69 to -0.12]), while MVAG values were also significantly lower at 12-month follow-up than baseline (*p*=0.021, *d_unb_*=-0.96 [-1.83 to -0.15]).

Knee Extension (KE) for non-dominant side (NDS)

For RTD_0–30_, both groups demonstrated significantly lower values at baseline than post-intervention (LAG: *p*<0.001, *d_unb_*=1.19 [0.70 to 1.76]; MVAG: *p*=0.001, *d_unb_*=0.53 [0.26 to 0.84]), six-month (LAG: *p*<0.001, *d_unb_*=0.87 [0.46 to 1.33]; MVAG: *p*=0.030, *d_unb_*=0.35 [0.12 to 0.62]), and 12-month follow-ups (LAG: *p*<0.001, *d_unb_*=0.92 [0.45 to 1.45]; MVAG: *p*=0.001, *d_unb_*=0.60 [0.27 to 0.98]). For RTD_0–60_, MVAG displayed significantly higher values at post-intervention compared to six-month follow-up (*p*=0.003, *d_unb_*=-0.45 [-0.75 to -0.19]), while both groups presented significantly lower values at baseline than post-intervention (LAG: *p*=0.001, *d_unb_*=0.78 [0.36 to 1.26]; MVAG: *p*<0.001, *d_unb_*=0.58 [0.26 to 0.94]). For RTD_0-80_, only the MVAG showed significantly higher values at 12-month follow-up than baseline (*p*=0.018, *d_unb_*=-0.54 [-0.99 to -0.13]), and post-intervention (*p*=0.016, *d_unb_*=-0.47 [-0.84 to -0.14]). For RTD_30–60_, only the LAG exhibited significantly higher values at baseline than post-intervention (*p*=0.048, *d_unb_*=-0.83 [-1.54 to -0.17]), six-month (*p*<0.001, *d_unb_*=-1.07 [-1.69 to -0.52]), and 12-month follow-ups (*p*=0.008, *d_unb_*=-1.17 [-1.96 to -0.45]). For RTD_30–80_, both groups displayed significantly higher values at baseline than six-month (LAG: *p*=0.003, *d_unb_*=-1.17 [-1.88 to -0.55]; MVAG: *p*=0.009, *d_unb_*=-0.96 [-1.75 to -0.26]), and 12-month follow-ups (LAG: *p*<0.001, *d_unb_*=-1.66 [-2.48 to -0.97]; MVAG: *p*<0.001, *d_unb_*=-1.51 [-2.51 to -0.64]). In addition, LAG presented significantly higher values at baseline compared to post-intervention (*p*=0.006, *d_unb_*=-1.16 [-1.95 to -0.45]). Lastly, for RTD_60–80_, while both groups showed significantly higher values at baseline than post-intervention (LAG: *p*=0.030, *d_unb_*=-1.07 [-1.81 to -0.38]; MVAG: *p*=0.032, *d_unb_*=-0.89 [-1.71 to -0.14]), the MVAG presented significantly higher values at baseline than six-month (*p*=0.039, *d_unb_*=-0.83 [-1.67 to -0.05]), and 12-month follow-ups (*p*<0.001, *d_unb_*=-1.29 [-2.19 to -0.49]).

Knee Flexion (KF) for dominant side (DS)

For RTD_0–30_, both groups displayed significantly lower values at baseline compared to six-month (LAG: *p<*0.001, *d_unb_*=1.39 [0.74 to 2.13]; MVAG: *p*<0.001, *d_unb_*=0.79 [0.34 to 1.31]), and 12-month follow-ups (LAG: *p*=0.010, *d_unb_*=0.98 [0.37 to 1.66]; MVAG: *p*=0.001, *d_unb_*=0.74 [0.29 to 1.24]). For RTD_0–60_, both groups displayed significantly lower values at baseline than six-month follow-up (LAG: *p*=0.032, *d_unb_*=0.97 [0.43 to 1.57]; MVAG: *p*=0.001, *d_unb_*=0.70 [0.21 to 1.25]). The MVAG values were also significantly lower at baseline compared to 12-month follow-up (*p*=0.001, *d_unb_*=0.65 [0.25 to 1.09]). Similarly, for RTD_0-80_, both groups demonstrated significantly lower values at baseline than six-month follow-up (LAG: *p*=0.047, *d_unb_*=1.09 [0.56 to 1.69]; MVAG: *p*=0.001, *d_unb_*=0.84 [0.25 to 1.49]), and the MVAG values were also significantly lower at baseline than 12-month follow-up (*p*=0.004, *d_unb_*=0.60 [0.15 to 1.09]).

Knee Flexion (KF) for non-dominant side (NDS)

For RTD_0–30_, both groups displayed significantly lower values at baseline compared to post-intervention (LAG: *p*=0.001, *d_unb_*=1.28 [0.61 to 2.02]; MVAG: *p*=0.006, *d_unb_*=0.68 [0.21 to 1.20]), and 12-month follow-up (LAG: *p*=0.006, *d_unb_*=1.22 [0.71 to 1.82]; MVAG: *p*=0.010, *d_unb_*=0.66 [0.08 to 1.28]). For RTD_0–60_, both groups demonstrated significantly lower values at baseline compared to post-intervention (LAG: *p*=0.009, *d_unb_*=0.92 [0.28 to 1.63]; MVAG: *p*=0.050, *d_unb_*=0.49 [0.16 to 0.88]), and 12-month follow-up (LAG: *p*=0.001, *d_unb_*=1.28 [0.69 to 1.96]; MVAG: *p*=0.003, *d_unb_*=0.62 [0.17 to 1.12]). Lastly, for RTD_0-80_, both groups presented significantly lower values at baseline than 12-month follow-up (LAG: *p*=0.001, *d_unb_*=1.29 [0.62 to 2.06]; MVAG: *p*=0.030, *d_unb_*=0.47 [0.09 to 0.88]), while the LAG values were also significantly higher at post-intervention than baseline (*p*=0.005, *d_unb_*=1.01 [0.29 to 1.79]).

# **Supplementary Table S1:** Physical activity levels after the cessation of the 16-week high resistance training program.

| **Physical Activity Levels** | **Intervention** | | **Follow-up** | |
| --- | --- | --- | --- | --- |
|  | **M0 – Pre**  **N (%)** | **M1 – Post**  **N (%)** | **M2 – 6-Month**  **N (%)** | **M3 – 12-Month**  **N (%)** |
| Light | 11 (30.6) | 0 (0) | 10 (27.8) | 20 (55.6) |
| Moderate | 25 (69.4) | 4 (11.1) | 22 (61.1) | 11 (30.6) |
| Vigorous | 0 (0) | 32 (88.9) | 4 (11.1) | 5 (13.9) |

**Supplementary Table S2:** Distribution of self-reported physical activities at 12-month follow-up: light *vs.* moderate-to-vigorous intensity.

| **Groups** | **Activities self-reported** |
| --- | --- |
| LAG | 1 Participant – Occasionally walks at low intensity and yoga therapy  2 Participants – Once-a-week outdoor activity  4 Participants – Biweekly hydro gymnastic  6 Participants – Occasionally walks at low intensity  7 Participants – Did not perform any physical activity |
| MVAG | 1 Participant – 2–3 sessions per week of moderate-intensity continuous training, high-intensity interval training, and 2–3 walks longer than 40 min  1 Participant – Resistance training at least 2 times per week  2 Participants – Walk every day and biweekly hydro gymnastics  5 Participants – Walks longer 40 min every day  7 Participants – 2–3 sessions per week of moderate-intensity continuous training and high-intensity interval training |
| Abbreviatures: LAG, light activity group; MVAG, moderate-to-vigorous activity group. | |

**Supplementary Table S3:** Changes in physical activity over the study period.

| **Measures** | **Groups** | **Intervention** | | **Follow-up** | | ***Time Effect*** | ***Interaction effect Within groups*** | ***Interaction effect Between groups*** |
| --- | --- | --- | --- | --- | --- | --- | --- | --- |
|  |  | **M0**  **Pre** | **M1**  **Post** | **M2**  **6-Month** | **M3**  **12-Month** |  |  |  |
| Total Activity  (days) | LAG **^a,c,d,e,f^** | 5.35 ± 2.01 | 6.95 ± 0.22 | 5.70 ± 2.03 | 2.90 ± 1.48 **†** | F=14.403¥  ***p*<0.001**  η²_p_=0.298§ | F=15.512¥  ***p*<0.001**  η²_p_=0.313§ | F=10.654  ***p*=0.003**  η²_p_=0.239§ |
|  | MVAG | 5.75 ± 1.88 | 6.88 ± 0.50 | 5.56 ± 1.75 | 6.62 ± 0.72 |  |  |  |
|  | TS **^a,d,e^** | 5.53 ± 1.93 | 6.92 ± 0.37 | 5.64 ± 1.89 | 4.56 ± 2.22 |  |  |  |
| Total Activity  (minute/week) | LAG **^a,d,e^** | 71.25 ± 42.36 | 131.25 ± 29.46 | 65.75 ± 38.33 | 54.25 ± 33.02 **†** | F=29.331  ***p*<0.001**  η²_p_=0.463§ | F=3.009  ***p*=0.034**  η²_p_=0.081* | F=6.602  ***p*=0.015**  η²_p_=0.163§ |
|  | MVAG **^a,d,e^** | 74.38 ± 29.71 | 137.50 ± 29.72 | 86.25 ± 44.10 | 99.06 ± 34.36 |  |  |  |
|  | TS **^a,d,e,^** | 72.64 ± 36.81 | 134.03 ± 29.32 | 74.86 ± 41.69 | 74.17 ± 40.09 |  |  |  |
| MVPA (MET/minute/week) | LAG **^a,d,e^** | 470.00 ± 404.32 | 1991.00 ± 444.21 | 591.00 ± 602.08 | 231.00 ± 302.03**†** | F=91.126  ***p<*0.001**  η²_p_=0.728§ | F=7.368  ***p<*0.001**  η²_p_=0.178§ | F=18.252  ***p<*0.001**  η²_p_=0.349§ |
|  | MVAG **^a,c,d,e,f^** | 596.25 ± 420.17 | 2226.25 ± 513.33 | 706.25 ± 493.41 | 1225.00 ± 579.68 |  |  |  |
|  | TS **^a,d,e^** | 526.11 ± 410.43 | 2095.56 ± 483.84 | 642.22 ± 551.81 | 672.78 ± 666.68 |  |  |  |
| Walking  (MET/minute/week) | LAG | 603.90 ± 585.56 | 501.60 ± 529.69 | 1110.45 ± 1899.67 | 222.75 ± 219.39**†** | F=2.001¥  ***p*=0.137**  η²_p_=0.056# | F=1.565¥  *p*=0.213  η²_p_=0.044* | F=1.007  *p*=0.323  η²_p_=0.029 |
|  | MVAG | 1027.13 ± 1766.53 | 1103.44 ± 1397.32 | 662.06 ± 730.83 | 419.72 ± 310.68 |  |  |  |
|  | TS | 792.00 ± 1252.61 | 769.08 ± 1039.75 | 911.17 ± 1496.33 | 310.29 ± 278.11 |  |  |  |
| Sitting Time  (hours) | LAG **^a,d,e,f^** | 4.95 ± 1.82 | 3.80 ± 1.06 **†** | 4.85 ± 1.89 | 5.80 ± 1.61**†** | F=19.010  ***p*<0.001**  η²_p_=0.359§ | F=1.331  *p*=0.268  η²_p_=0.038# | F=3.360  *p*=0.076  η²_p_=0.090* |
|  | MVAG **^a,d,e^** | 4.63 ± 1.03 | 3.13 ± 0.81 | 4.31 ± 1.58 | 4.56 ± 0.89 |  |  |  |
|  | TS **^a,d,e^** | 4.81 ± 1.51 | 3.50 ± 1.00 | 4.61 ± 1.76 | 5.25 ± 1.46 |  |  |  |
| Significant differences between periods are highlighted in bold (*p*≤0.050). Abbreviations: LAG, light activity group; MVAG, moderate-to-vigorous activity group; TS, total sample; MET, metabolic equivalent task; PA, physical activity; MVPA, sum of minutes spent in moderate and vigorous activity.  ¥, Greenhouse‒Geisser correction.  Significant differences:  a, pre-intervention *vs.* post-intervention; b, pre-intervention *vs.* 6-month follow-up; c, pre-intervention *vs.* 12-month follow-up; d, post-intervention *vs.* 6-month follow-up;  e, post-intervention *vs.* 12-month follow-up; f, 6-month follow-up *vs.* 12-month follow-up.  †, between groups at that assessment point.  η²_p_ values thresholds:  #, small effect: 0.010 to 0.059; *, medium effect: 0.060 to 0.140; §, large effect large: > 0.140. | | | | | | | | |


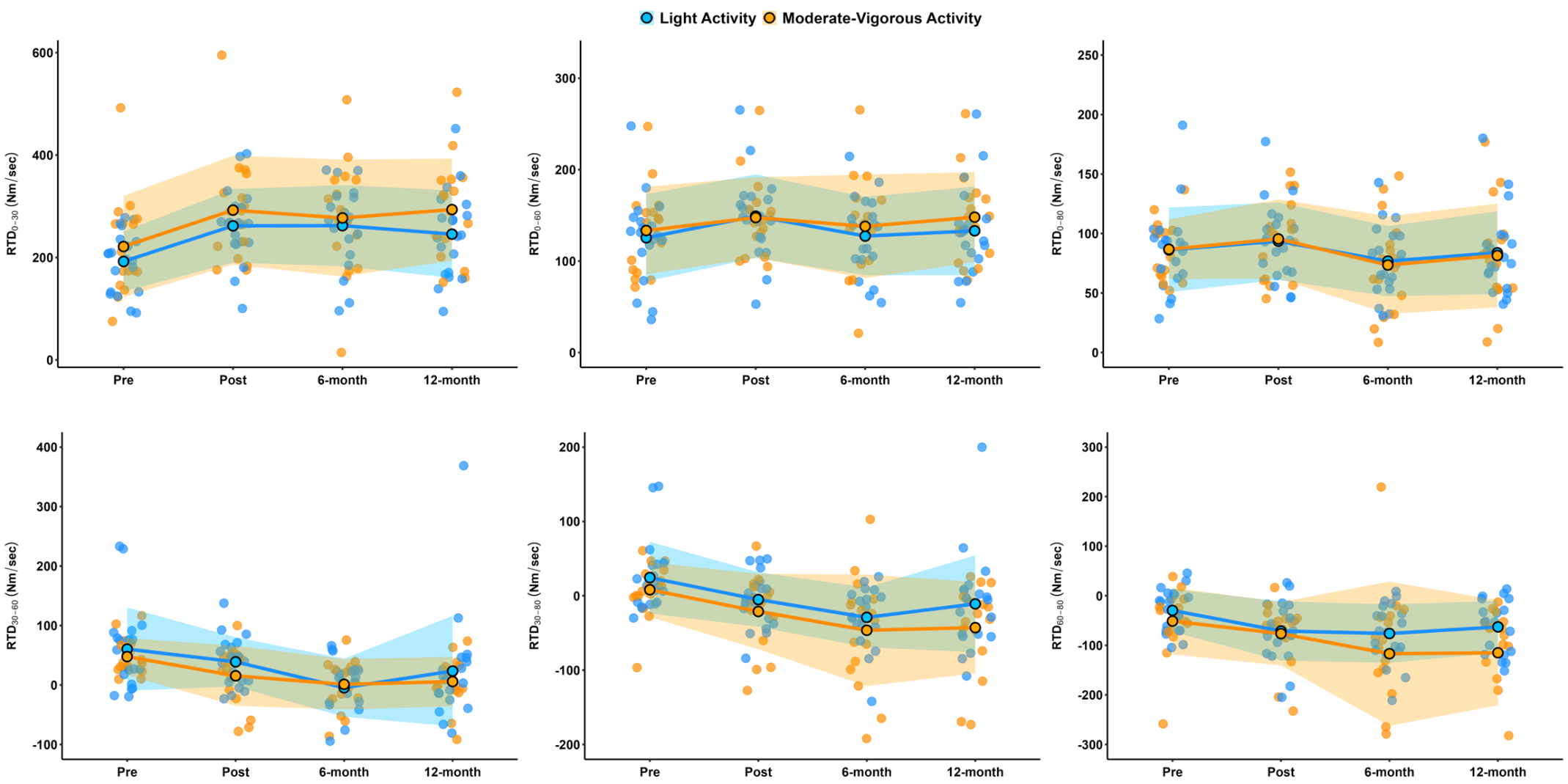


**Supplementary Fig. S1:** Rate of torque development time intervals_(0–30 ms, 0–60 ms, 0–80 ms, 30–60 ms, 30–80 ms, and 60–80 ms)_ for knee extension on the dominant side in the light activity and moderate-to-vigorous activity groups. Mean values are represented by solid lines and filled dots, with shaded areas indicating the standard deviation.


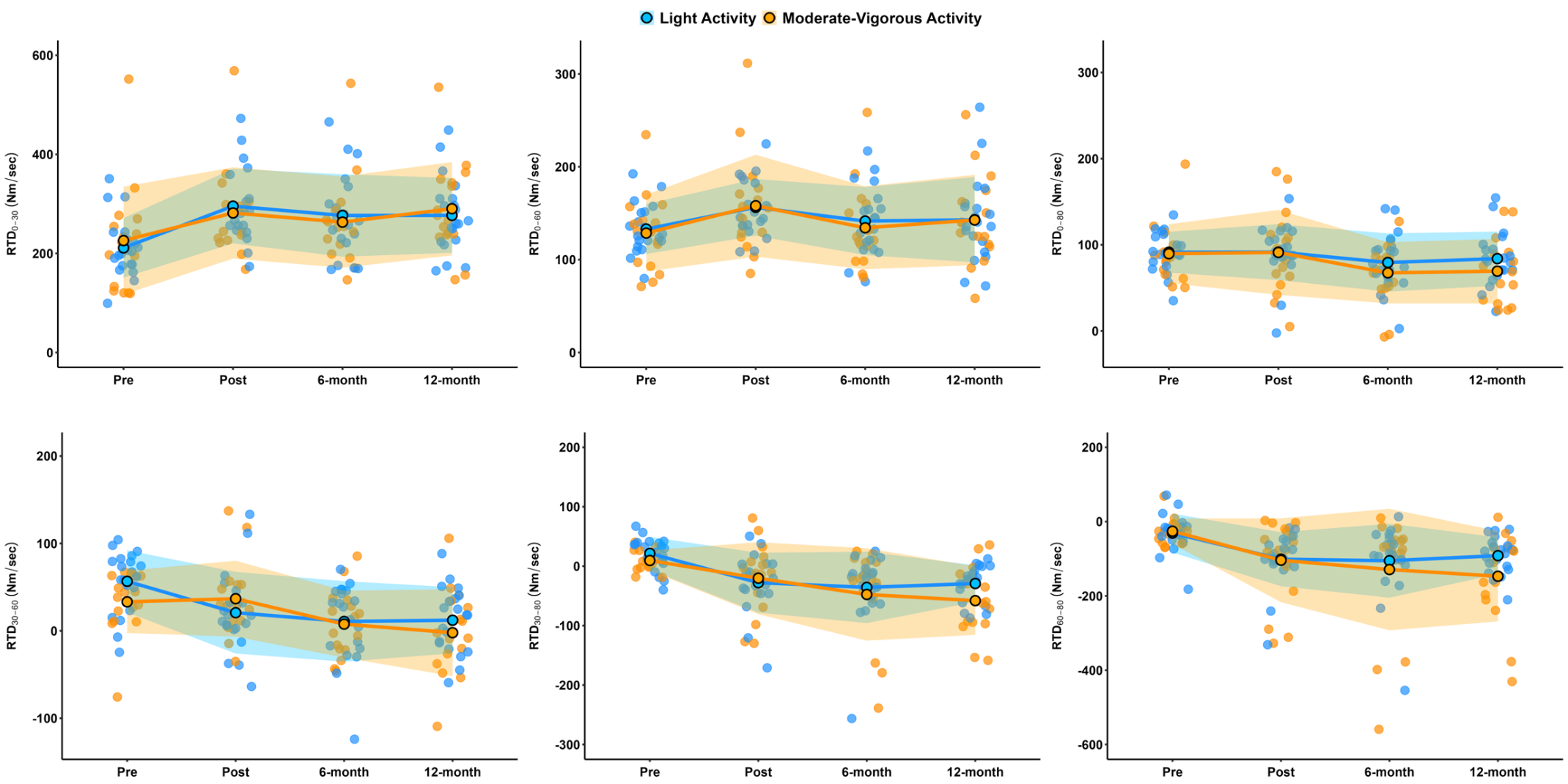


**Supplementary Fig. S2:** Rate of torque development time intervals_(0–30 ms, 0–60 ms, 0–80 ms, 30–60 ms, 30–80 ms, and 60–80 ms)_ for knee extension on the non-dominant side in the light activity and moderate-to-vigorous activity groups. Mean values are represented by solid lines and filled dots, with shaded areas indicating the standard deviation.


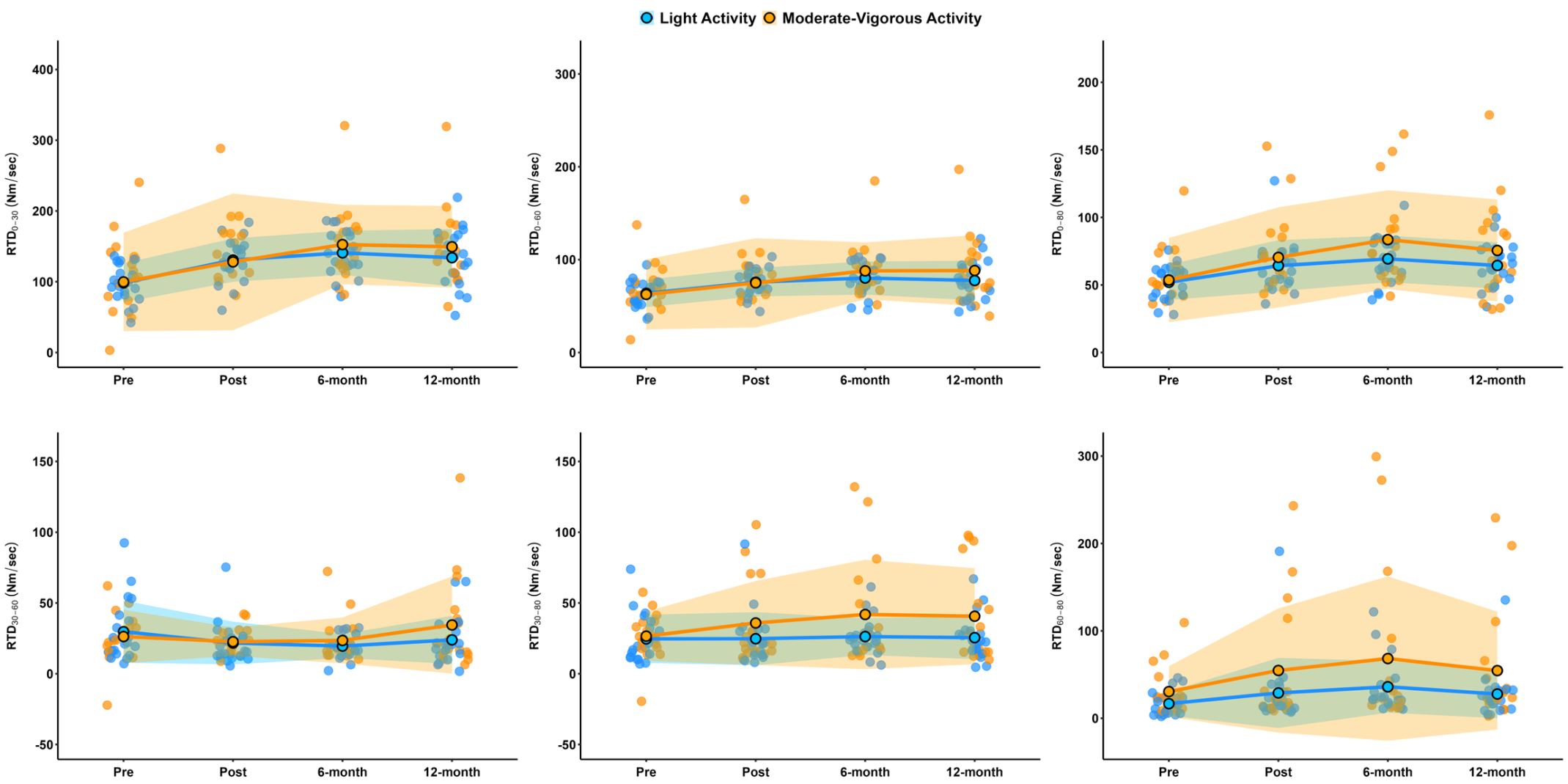


**Supplementary Fig. S3:** Rate of torque development time intervals_(0–30 ms, 0–60 ms, 0–80 ms, 30–60 ms, 30–80 ms, and 60–80 ms)_ for knee flexion on the dominant side in the light activity and moderate-to-vigorous activity groups. Mean values are represented by solid lines and filled dots, with shaded areas indicating the standard deviation.


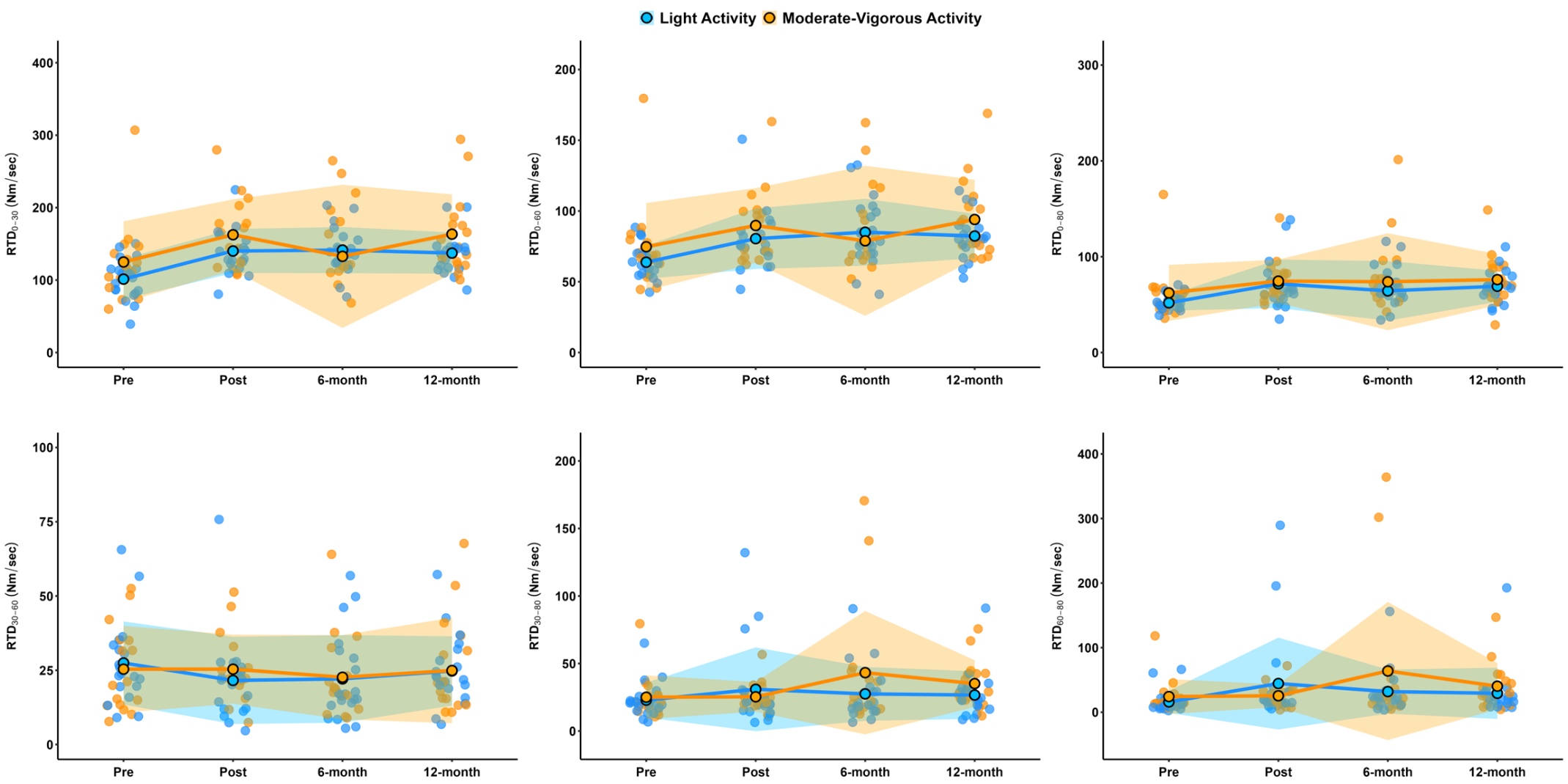


Supplementary Fig. S4: Rate of torque development time intervals_(0–30 ms, 0–60 ms, 0–80 ms, 30–60 ms, 30–80 ms, and 60–80 ms)_ for knee flexion on the non-dominant side in the light activity and moderate-to-vigorous activity groups. Mean values are represented by solid lines and filled dots, with shaded areas indicating the standard deviation.
